# Enhanced Osteoporosis Detection Using Artificial Intelligence: A Deep Learning Approach to Panoramic Radiographs with an Emphasis on the Mental Foramen

**DOI:** 10.3390/medsci12030049

**Published:** 2024-09-20

**Authors:** Robert Gaudin, Wolfram Otto, Iman Ghanad, Stephan Kewenig, Carsten Rendenbach, Vasilios Alevizakos, Pascal Grün, Florian Kofler, Max Heiland, Constantin von See

**Affiliations:** 1 Charité—Universitätsmedizin Berlin, Corporate Member of Freie Universität Berlin and Humboldt Universität zu Berlin, Department of Oral and Maxillofacial Surgery, Augustenburger Platz 1, 13353 Berlin, Germany; robert-andre.gaudin@charite.de (R.G.); wolfus.otto@charite.de (W.O.); iman.ghanad@charite.de (I.G.); stephan.kewenig@charite.de (S.K.); carsten.rendenbach@charite.de (C.R.); max.heiland@charite.de (M.H.); 2 Berlin Institute of Health, Charité—Universitätsmedizin Berlin, 10117 Berlin, Germany; 3 Center for Digital Technologies in Dentistry and CAD/CAM, Danube Private University, 3500 Krems an der Donau, Austria; constantin.see@dp-uni.ac.at; 4 Center for Oral and Maxillofacial Surgery, Faculty of Medicine/Dental Medicine, Danube Private University, 3500 Krems an der Donau, Austria; pascal.gruen@dp-uni.ac.a; 5 Helmholtz AI, Helmholtz Zentrum München, Ingostaedter Landstrasse 1, 85764 Oberschleissheim, Germany; florian.kofler@tum.de; 6 TUM—Neuroimaging Center, Klinikum Rechts der Isar, Technical University of Munich, 81675 Munich, Germany

**Keywords:** osteoporosis detection, deep learning, panoramic radiographs, convolutional neural network (CNN), early diagnostic tool

## Abstract

Osteoporosis, a skeletal disorder, is expected to affect 60% of women aged over 50 years. Dual-energy X-ray absorptiometry (DXA) scans, the current gold standard, are typically used post-fracture, highlighting the need for early detection tools. Panoramic radiographs (PRs), common in annual dental evaluations, have been explored for osteoporosis detection using deep learning, but methodological flaws have cast doubt on otherwise optimistic results. This study aims to develop a robust artificial intelligence (AI) application for accurate osteoporosis identification in PRs, contributing to early and reliable diagnostics. A total of 250 PRs from three groups (A: osteoporosis group, B: non-osteoporosis group matching A in age and gender, C: non-osteoporosis group differing from A in age and gender) were cropped to the mental foramen region. A pretrained convolutional neural network (CNN) classifier was used for training, testing, and validation with a random split of the dataset into subsets (A vs. B, A vs. C). Detection accuracy and area under the curve (AUC) were calculated. The method achieved an F1 score of 0.74 and an AUC of 0.8401 (A vs. B). For young patients (A vs. C), it performed with 98% accuracy and an AUC of 0.9812. This study presents a proof-of-concept algorithm, demonstrating the potential of deep learning to identify osteoporosis in dental radiographs. It also highlights the importance of methodological rigor, as not all optimistic results are credible.

## 1. Introduction

Osteoporosis, a systemic skeletal disorder marked by reduced bone mass, is anticipated to affect 60% of women over the age of 50 years by 2040 [1]. The current gold standard for osteoporosis detection is a dual-energy X-ray absorptiometry (DXA) scan, which is only available in specialized osteoporosis centers and typically employed post-fracture [2,3]. In addition to DXA measurements, the diagnosis of osteoporosis takes a history of fragility fractures and clinical risk factors such as age, gender, and family history into account [1]. Given that osteoporosis-related fractures impose a financial burden on the health-care system amounting to double-digit billions of dollars worldwide, there is a critical demand for tools that are capable of early detection [4]. However, age-related changes in bone density necessitate a tailored interpretation of DXA results [5]. As individuals age, their bone density naturally decreases because of factors such as hormonal changes and decreased physical activity. DXA scans provide valuable insights into the longitudinal alterations in bone density, thereby enabling proactive interventions aimed at attenuating bone demineralization and diminishing the likelihood of fractures in aging cohorts [6]. Consequently, the interpretation of cross-sectional investigations and individual longitudinal diagnoses requires meticulous consideration of each case’s unique circumstances. Generalizations made within mathematical models are subject to clinical controversy and warrant thorough discussion [7]. A few studies have been published on the alternative detection option for osteoporosis, including magnetic resonance imaging (MRI), optical coherence tomography (oCT), and quantitative computed tomography (qCT), which appear to be viable and may offer cost and time savings [8,9,10].

Nevertheless, given the significant impact of osteoporosis-related fractures on the health-care system caused by late detection, there is an urgent need for tools that enable early detection [11]. The solutions discussed previously do not meet the requirements for effective early detection screening, underscoring a significant medical need for the development of such technologies.

Panoramic radiography (PR) is a routine component of dental evaluations and gives a 2D overview of the dentition, the jaw, and the hard surrounding tissue. This may be conducted annually and presents a viable medium for such early detection efforts [12].

Numerous studies have applied PR for osteoporosis detection, supporting its potential as an effective screening tool [13,14,15,16]. In this context, specific measures that are derivable from panoramic radiographs (PRs) have been developed to assist in osteoporosis identification. These measures include three primary indices: the Mandibular Cortical Index (MCI), which assesses the shape of the mandibular cortex; the mandibular cortical width (MCW), which evaluates the ratio of the mandibular cortical width to the cortical height; and the Panoramic Mandibular Index (PMI), which is calculated as the ratio of the thickness of the mandibular cortex to the distance between the mental foramen and the inferior mandibular cortex [17]. Notably, the MCI, also referred to as the Klemetti Index (KI), was introduced by Klemetti in 1993 [18] and has since become the most frequently utilized indices for this purpose [19]. Nevertheless, these indices are rarely used in the clinical workflow, since they must be manually calculated, are prone to errors, and are time-consuming. Comparing it to a gold standard technique such as DXA has proven it to be unreliable [20,21]. Various studies of osteoporosis detection in PRs using a deep convolutional neural network (CNN) have been published recently, with promising outcomes [2,22]. However, upon closer examination of the methodology and design of these studies, factors such as age, overlying structures, gender, disease severity, and radiographic technique may have influenced the radiographs’ ability to accurately detect osteoporosis-related changes, consequently affecting the algorithms’ performance [23,24]. These variables were not adequately addressed in those studies, highlighting significant flaws in the application of PRs for osteoporosis detection and emphasizing the necessity for cautious interpretation. Furthermore, the methods of recent studies were not transparent. The aim of this study is to rectify these shortcomings of past studies and develop a reliable artificial intelligence (AI) application for the accurate detection of osteoporosis in PRs.

## 2. Materials and Methods

This study was conducted according to the guidelines of the Medical World Association (Declaration of Helsinki). Ethical approval was granted by the Institutional Review Board of the Charité ethics committee (EA2/089/22). The checklist for AI research in dentistry of the ITU/WHO focus group “Artificial Intelligence for Health (AI4H)” was consulted for the reporting in this study [25].

Data

This study retrospectively included patients who were diagnosed with osteoporosis at Danube Private University. Each patient underwent a DXA scan to assess their bone mineral density (BMD). Patients with a BMD T-score above −1 were classified as healthy, while those with a BMD T-score below −2.5 were diagnosed with osteoporosis (group A). PRs were retrieved from the medical records of each patient within 24 h following the DXA scan. After the radiographs of the osteoporosis patient group were collected, a second group of patients, related to the first group’s age and gender, was used as the control group (group B). Another healthy group of a non-correlating age (young patients) and gender distribution was also collected for validation purposes.

Data Preparation and Model Training

A total of 250 PRs with osteoporosis and a control group of 250 PRs without osteoporosis were automatically cropped to the region of interest of the mental foramen after the annotation of 60 images in the CvatAI annotator (https://www.cvat.ai/) using the YoloV8 code. The region of interest, ROI, of the mental foramen was chosen based on the KI forecasting the most promising results [18]. Subsequently, all images were visually inspected by an experienced investigator to identify any missing or improperly cropped images in the designated area. In total, a dataset of 500 grayscale images (comprising left and right views of the foramen and its surrounding area) was prepared meticulously for each group. The mental foramen was selected as the region of interest based on the aforementioned indices, which incorporate this anatomical structure. The cropped PRs with osteoporosis were randomly divided into three splits (70% train, 15% test, 15% validation). The control PRs without osteoporosis were sampled in accordance with the gender and age distribution of the training set (age = 54 ± 16 a, female/male ratio = 3/1). The validation split was used to select an optimal model performance during training and hyperparameter selection, while the held-out test split was used to evaluate the model’s performance after training and hyperparameter selection.

For data preparation, the cropped PR images were augmented using random rotation with 10° and color-jitter (brightness = 0.2, hue = 0.1, contrast = 0.3, saturation = 0.3) and cropped and vertically flipped at random. Afterward, the data were scaled to 224 × 224 and normalized.

The model optimization employed the SGD optimizer with an initial learning rate of 0.001 and a batch size of 16. To mitigate overfitting, an L2 lambda regularization strength of 1 × 10^−6^ was applied. The model architecture was based on the pretrained Densenet201 network, which was adapted for binary classification output. After adjusting hyperparameters, the model underwent training for 150 epochs. Implementation was carried out using the PyTorch code, and training was performed on a single V100 GPU (NVIDIA).

**Figure 1:**
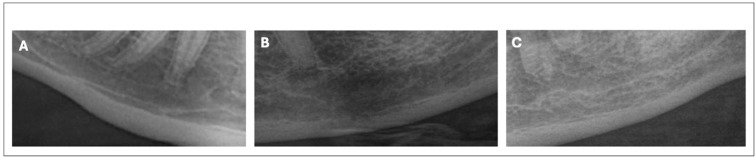
Automatically rectangular images, cropped to the region of interest of the mental foramen: (**A**) young patients, (**B**) old patients without osteoporosis, and (**C**) old patients with osteoporosis.

Furthermore, 500 cropped PRs of young patients under the age of 30 years without osteoporosis were used for the same model, performed with the same hyperparameter (age = 24 ± 6 a, female/male ratio = 3/1); see Figure 1.

Statistical Analysis

The accuracy of osteoporosis detection in the foramen regions involved the processing of the cropped region using its classifier. Based on these predictions, performance metrics were computed, including precision on the validation set, as well as a confusion matrix for the comparison of the osteoporosis group versus the control group of the same age and the comparison of the osteoporosis group versus the young control group.

## 3. Results

**Figure 2:**
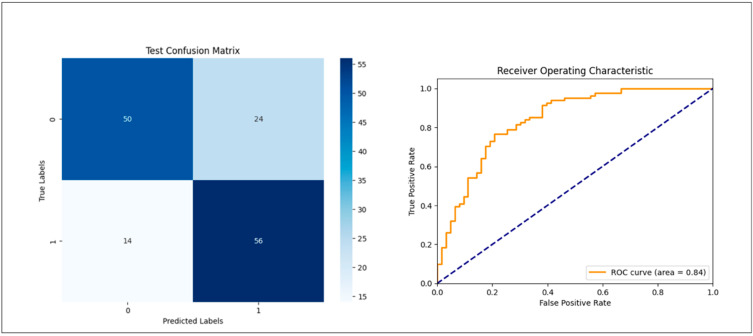
Confusion matrix illustrating, on the left, the detection results of osteoporosis based on PR for an untouched validation set after training and hyperparameter selection and, on the right, the receiver operating characteristic (ROC) curve.

**Figure 3:**
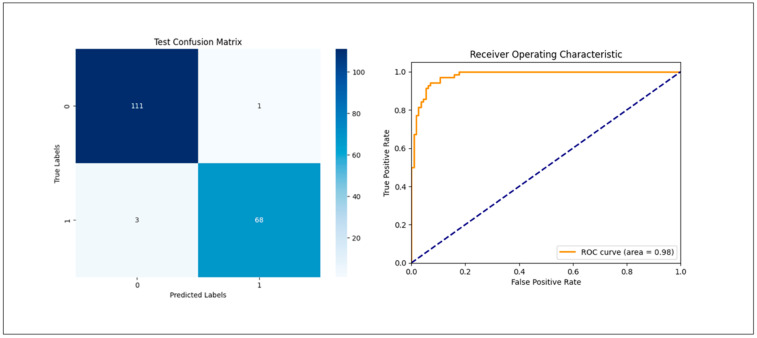
Confusion matrix illustrating, on the left, the detection results of osteoporosis based on PR for an untouched validation set of the osteoporosis group versus a young control group and, on the right, the receiver operating characteristic (ROC) curve, indicating that the high accuracy is due to the recognition of a young-versus-old bone structure, making the results misleading.

The detection accuracy and area under the curve (AUC) were calculated for the datasets. In Figure 2 and Figure 3, the confusion matrix illustrates the detection results of osteoporosis on PR. 

The model achieved a precision of 73.6% and an F1 score of 0.74 for the validation accuracy in the group comparing osteoporosis patients with the age-matched control group (Table 1). The AUC was 0.84. In the group comparing osteoporosis patients with the young control cohort, the model demonstrated an accuracy of 97.8% and an AUC of 0.98, with an F1 score of 0.97 (Table 2).

The correlating confusion matrices are shown in Table 1 and Table 2.

## 4. Discussion

This study demonstrated the capability of a deep learning algorithm to detect osteoporosis indicators in dental radiographs. It demonstrated the model’s effective performance in distinguishing osteoporosis patients from control groups of varying ages. The findings underscore its robustness in diagnostic accuracy and discrimination ability, emphasizing its potential utility in clinical practice for diverse demographic profiles. Within these models, significant differences in AI-assisted osteoporosis diagnosis outcomes emerged, primarily stemming from discrepancies in data selection and annotation methodologies [2,22]. For instance, Sukegawa et al. [2], utilizing a meticulously curated dataset with comprehensive annotations, achieved commendable accuracy rates in identifying osteoporotic markers from dental radiographs. Conversely, Lee et al. [22], using inadequately annotated data with limited diversity, produced inferior results, highlighting the significant impact of data quality on AI performance.

In this case, the inclusion of young patients in the dataset falsely improves the algorithm’s performance, as our study demonstrated through our calculations. Comparing the model to 224 images of young patients, the algorithm performed with a 97.81% accuracy. The performance of AI systems is significantly affected not only by data acquisition but also by factors such as the network architecture and hyperparameters during training and validation. This complexity poses challenges in assessing the reliability of reported results. In the field of automated data detection, the importance of data selection and annotation cannot be overstated. They form the basis upon which AI models are constructed, directly influencing their performance and outcomes. Numerous studies have emphasized the pivotal role of data quality in AI applications [26,27]. The choice of data, their relevance, and the accuracy of annotation are pivotal factors that influence the efficacy of AI algorithms. In medical imaging, where precision and reliability are paramount, the impact of data selection and annotation is particularly pronounced. This investigation also reveals several limitations. The applications of PRs are not considered the gold standard for osteoporosis detection. The small sample size increases the risk of overfitting, potentially affecting the model’s performance when applied to new datasets. The use of only one type of radiograph further limits the model’s generalizability, as it may not perform as well with other imaging modalities. Additionally, the dataset is derived from a single institution, which may introduce biases that are specific to that population and reduce the external validity of the findings. As a result, the model’s applicability to broader clinical settings and diverse patient populations remains uncertain. Future studies should aim to validate these findings using larger, multi-institutional datasets and a variety of radiographic types to improve generalizability and robustness. Enhancing the data volume through multi-center collaborative efforts could elevate the accuracy and generalizability of the model’s diagnostic classification. Another limitation concerns the specific types of models evaluated. This research assessed the performance of YOLOv8 at various depths. Discovering an architecture with fewer parameters that maintains or improves performance could enhance its applicability by reducing computational costs. YOLOv8 is an object detection model that has not been specifically developed on medical images. Future research should focus on identifying an architecture that is optimal for different image qualities and patient demographic variables. Lastly, the manual cropping of images to position the mental foramen within the mandible based on the common indices introduces a potential bias in preoperative preparation. This underscores the need for refined image preparation techniques in future studies.

In the future, it will be essential to evaluate this model alongside medical professionals to assess whether their diagnostic accuracy improves when they utilize regions that are highlighted by deep learning techniques. Conducting such comparisons will aid in enhancing the development and application of deep learning methodologies. 

Additional studies are required to ascertain the specific areas within a PR that the model identifies as significant, considering that the image encompasses various anatomical structures. Moreover, it is crucial to investigate the model’s adaptability and performance in terms of generalizability using datasets from different institutions.

## 5. Conclusions

This research showcased a proof-of-concept algorithm that highlights the potential of deep learning in identifying osteoporosis indicators in dental radiographs. Furthermore, our thorough examination of existing algorithms revealed that not all optimistic outcomes hold credibility under scrutiny of methodological integrity. Despite promising results, several limitations must be considered. The reliance on a small, single-institution dataset increases the risk of overfitting and limits generalizability. Furthermore, the exclusive use of PRs, which are not the gold standard for osteoporosis detection, and the need for manual image preparation introduce potential biases. Future research should focus on validating these findings with larger, multi-institutional datasets and exploring models that are better suited for medical imaging. Additionally, integrating deep learning techniques into clinical practice will require further evaluation alongside expert radiologists to enhance the diagnostic accuracy and practical applicability.

## Figures and Tables

**Table 1:** Confusion matrix for the comparison of the osteoporosis group versus the control group of the same age.

Model for Osteoporosis Group versus Control of Same Age		
Measure	Value	Derivations
Sensitivity	0.7000	TPR = TP / (TP + FN)
Specificity	0.7813	SPC = TN / (FP + TN)
Precision	0.8000	PPV = TP / (TP + FP)
Negative Predictive Value	0.6757	NPV = TN / (TN + FN)
False Positive Rate	0.2188	FPR = FP / (FP + TN)
False Discovery Rate	0.2000	FDR = FP / (FP + TP)
False Negative Rate	0.3000	FNR = FN / (FN + TP)
Accuracy	0.7361	ACC = (TP + TN) / (P + N)
F1 Score	0.7467	F1 = 2TP / (2TP + FP + FN)
Matthews Correlation Coefficient	0.4785	TP × TN − FP × FN / sqrt((TP + FP) × (TP + FN) × (TN + FP) × (TN + FN))

**Table 2:** Confusion matrix for the comparison of the osteoporosis group versus the control group of the same age and the comparison of the osteoporosis group versus the young control group.

Model for Osteoporosis Group versus Control of Young Age		
Measure	Value	Derivations
Sensitivity	0.9855	TPR = TP / (TP + FN)
Specificity	0.9737	SPC = TN / (FP + TN)
Precision	0.9577	PPV = TP / (TP + FP)
Negative Predictive Value	0.9911	NPV = TN / (TN + FN)
False Positive Rate	0.0263	FPR = FP / (FP + TN)
False Discovery Rate	0.0423	FDR = FP / (FP + TP)
False Negative Rate	0.0145	FNR = FN / (FN + TP)
Accuracy	0.9781	ACC = (TP + TN) / (P + N)
F1 Score	0.9714	F1 = 2TP / (2TP + FP + FN)
Matthews Correlation Coefficient	0.9540	TP × TN − FP × FN / sqrt((TP + FP) × (TP + FN) × (TN + FP) × (TN + FN))

## Data Availability

Data are contained within the article.
